# Numerical Simulations of Laser-Induced Shock Experiments on Graphite

**DOI:** 10.3390/ma14227079

**Published:** 2021-11-22

**Authors:** Alberto Morena, Lorenzo Peroni

**Affiliations:** Department of Mechanical and Aerospace Engineering, Politecnico di Torino, Corso Duca degli Abruzzi, 24, 10129 Turin, Italy

**Keywords:** finite element numerical simulation, laser–matter interaction, laser-induced shockwave, high-energy impact, graphite, beam intercepting devices

## Abstract

The development of particle accelerators with ever increasing energies is raising the standards of the structures which could interact with the particle beams. These structures could be subjected to strong shockwaves in accidental scenarios. In order to test materials in such conditions, one of the most promising techniques is the impact with high-power lasers. In view of the setting up of future experimental campaigns within the Petawatt High-Energy Laser for Heavy Ion Experiments (PHELIX), the present work aims at the development of a numerical approach for the simulation of graphite impacted by laser beams. In particular, the focus is on the spallation damage caused by shockwave reflection: a sufficiently intense laser beam could ablate the matter until plasma conditions, hence producing a shockwave which could travel inside the material and reach a free surface. A numerical model to properly describe the spall fragmentation of graphite has been calibrated on the basis of literature-available experimental data. The numerical approach is a ‘two-step’ procedure: the first step is the definition of the laser–matter interaction and the second one concerns the description of the shockwave evolution into matter. The simulations satisfactorily reproduce the dynamic response of graphite impacted by two different laser sources with various intensities, despite the difficulties of characterising a phenomenon which is extremely fast and chaotic.

## 1. Introduction

The investigation of the dynamic behaviour of materials is a strongly multi-disciplinary subject which is of paramount importance for several engineering applications. In particular, the analysis of shock loading conditions is fundamental in the civil, aerospace and military industry. Indeed, the response of matter to high strain rates is extremely different from quasi-static conditions; hence, the methods to study and define materials have been specifically adapted to this context.

Generally, the experiments deal with inducing a planar compression wave on the surface of the specimen and the aim is to cause the failure of the material. As a matter of fact, when the shockwave reaches the back of the target, it gets reflected as a tensile wave, thus generating a high strain rate tensile condition in the specimen. Therefore, the stress at which the material fractures is called spallation (or just spall) stress.

The shockwaves can be produced with different techniques, and the most used are hypervelocity impacts, explosives and energy depositions. Given the aim of this work, the focus is on energy depositions and more in detail on laser-driven shockwaves. The lasers have been considered and used as shockwave generators ever since the beginning of their history [1,2]. However, the laser applications are now numerous and some of the most widely known concern measurement systems. In fact, the laser interferometry is already strongly used not only in research, but also in industrial applications. Nowadays, the practice of inducing a shock through a laser is also commonly used in many industrial processes, such as laser shock peening, both with the aim of polishing/cleaning surfaces and of increasing the fatigue resistance, the LASAT adhesion tests, through which the bonding strength of adhesives is verified, as well as the cutting, engraving, welding and drilling of several materials [3,4,5,6].

The aim of the present work is to develop a reliable numerical model to predict the behaviour of graphite during a laser-driven shockwave impact. As a matter of fact, this study is performed in the framework of the H2020 European project ARIES, and in particular, it is correlated to the Work Package 17 activities [7]. The objective of the Work Package 17 is to develop and characterise novel materials based on graphitic and metallic matrices for collimators and shock absorbers of particle accelerators. Indeed, the highly energetic proton beams travelling in the modern particle accelerators, such as the CERN Large Hadron Collider (LHC) [8], brought the need of safely managing the possible catastrophic impacts of uncontrolled beams onto the machine devices. This urgence has recently further increased due to the study and development of even more energetic accelerators such as the High-Luminosity LHC upgrade (HL-LHC) [9], the Higher-Energy LHC [10] or the Future Circular Collider (FCC) [11]. For example, the HL-LHC upgrade is going to double the energy stored in the particle beam [12], and hence the C-C carbon composite actually used in the primary and secondary collimators will be limiting the capacities of the LHC [13]. In this challenging context, the CERN has launched a significative R&D program to search for novel materials with the purpose of taking advantage of the mechanical and thermal properties of graphite matrices [14,15,16] and comparing them with some adopted high temperature resistance metals [17,18,19,20,21]. In the last years, the materials that might be exposed to particle beams irradiation, which are usually referred to as Beam Intercepting Devices (BID), have been routinely tested in dedicated facilities such as the HiRadMat [22,23]. In this state-of-art facility, the materials used as BIDs are impacted by highly energetic proton beams coming directly from the CERN Super Proton Synchrotron. At the HiRadMat, both online measurements and post-mortem analysis are able to produce fundamental data for characterising novel materials and for validating numerical models.

In any case, the energy densities which can be achieved at the HiRadMat are only a fraction of those that might be reached in the accidental impacts between BIDs and the extremely energetic beams produced by future particle accelerators as the FCC [24,25]. Furthermore, test facilities to reach these extreme conditions will not be available for many years, at least until great particle accelerators such as the FCC will be commissioned.

For this reason, one of the objectives of the Work Package 17 of the ARIES project is to reach energy densities beyond those reachable at the HiRadMat through high-energy laser beams. In spite of the differences between laser and particle beams, the effects on the impacted materials have proven to be comparable [26]. At least locally, the beam coming from a laser generates the same energy deposition of a particle beam, hence inducing a quasi-instantaneous heating and eventually a change of phase of the lightened surface. The modern high-energy laser facilities are able to produce laser beams with intensities of several TW/cm^2^ with nanosecond pulses, thus heating the impacted surface until it reaches plasma conditions. From the successive plasma expansion, a shockwave is generated into the target, producing spallation conditions when it is reflected by the back surface.

In the framework of the ARIES project, an irradiation experiment of several novel materials is planned to be held at the PHELIX Z6 laser facility at the GSI of Darmstadt (Germany). The PHELIX Z6 laser was built to deliver energies up to 180 J with pulse durations of 5 nanoseconds and spot diameters of few millimetres [27]. The objective is to test several thin specimens of graphite of different grades, MoGr, CrGr, CFC and other innovative materials.

In view of this challenging experimental campaign, the present work has the aim of investigating the behaviour of graphite impacted by highly energetic laser beams through an extensive use of numerical simulations. Even though most of the experimental works concerning laser-induced shockwaves have been applied to metals, some efforts have also been made to characterise graphite [26,28]. Hence, taking into account the laser shots fired at the CEA research centre as the benchmark, some numerical simulations have been developed with the objective of extrapolating paramount information regarding the response of this porous material when impacted by laser beams. Furthermore, the results coming from the numerical simulations should give fundamental indications about the choice of the dimensions of the specimens, as well as of the quantities that could and should be monitored and of the most indicated instruments to measure them.

Given the widespread application of lasers, many efforts have been undertaken to predict the laser–matter interaction. Although some analytical models have been developed [29,30,31], the predominant method to predict these phenomena is through numerical simulations. In this context, specific radiation hydrodynamic codes were built [32,33,34]. Even though many of the hydrocodes are also able to describe the propagation of the wave generated from the plasma expansion and the free surface velocity profile, most of them are not able to simulate the fracture mechanisms inside the material and describe only 1D geometries. For this reason, one possible numerical approach is to simulate the laser energy deposition map with a specific radiation hydrocode and import this map into an explicit Finite Element Solver (FEM) with appropriate material models [35,36,37], such as Ansys Autodyn [38] or LS-DYNA [39].

## 2. Laser-Induced Shockwaves in Solids

During a shockwave, the stress waves greatly exceed the dynamic flow strength of the material, which means that the deviatoric component of stresses is negligible with respect to the hydrostatic. Hence, the material exhibits a fluid-like behaviour and the complete definition of matter is given by the Equation of State [40].

**Figure 1:**
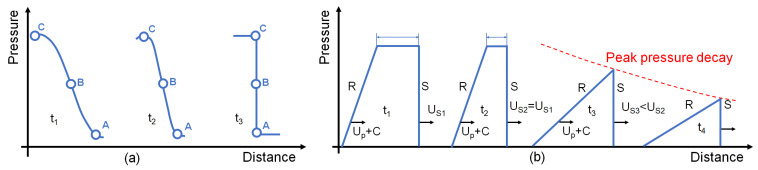
Graphical representation of: (**a**) the shockwave formation; (**b**) the propagation and attenuation of the shockwave, where S is the shock front and R is the release part of the shock, U_S_ is the shock front velocity, U_P_ is the particle velocity and C is the sound velocity at a particular pressure.

In general, a shockwave can be formed also, starting from quite smooth disturbances as shown in Figure 1a. This is due to the stiffening behaviour of the materials, which consists in the increasing of the sound velocity when the pressure increases. In fact, the front of the disturbance (point A) starts compressing the medium, and thus point C of the wave will be faster because it is travelling in a compressed matter. It means that the disturbance continues to steepen up until it reaches a shock front condition [40,41].

Indeed, there is a difference also between the velocities of the front and the release parts of the wave, given that the release is travelling in a medium which has a higher pressure and so a higher sound speed. This means that the release part of the wave is able to catch the shock front and to reduce the top as shown in Figure 1b; moreover, in some cases, it can also reduce the amplitude of the shockwave and the shock front speed.

**Figure 2:**
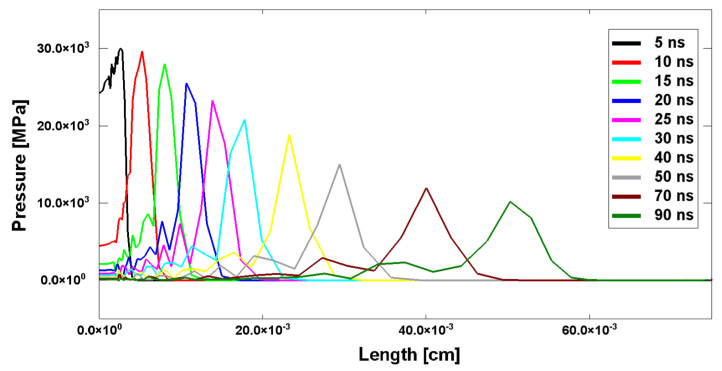
Helios hydrodynamic simulation of the evolution of a laser-induced shockwave inside the target for the shot S7 of the experimental case study explained below.

This feature is particularly relevant when dealing with shockwaves generated by laser pulses. As highlighted by the results reported in Figure 2, which belong to a hydrodynamic simulation of a laser-induced shockwave propagation, the wave has a triangular shape with the release part just behind the shock front; therefore, the peak begins to be attenuated almost immediately.

**Figure 3:**
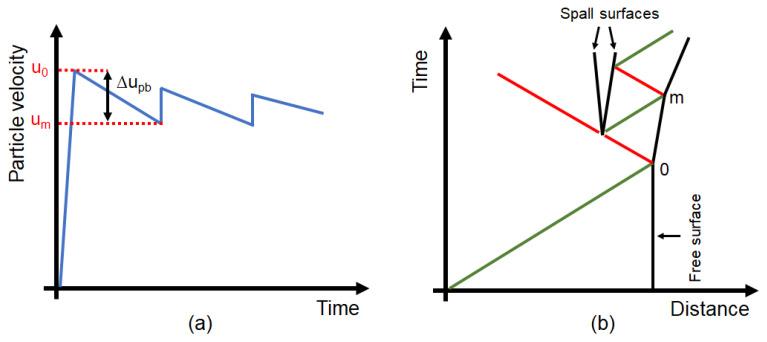
(**a**) Representation of the free surface velocity history. (**b**) Distance–time diagram of the compressive (green) and tensile (red) waves trajectories.

When the disturbance arrives at a free surface it gets reflected with a change of sign; that is, that the compression wave starts to travel backwards as a tensile wave. The dynamic fracture that occurs from the reflection of a shockwave is called a spall fracture, or just spall. In order to define the spall tensile stress, many strategies have been developed and the most used deal with the measurement of the rear surface velocity. In fact, when the front of the shock reaches the back surface, the velocity of that surface goes from zero to $u_{0}$. Whereas the release wave following the shock front is responsible for the deceleration of the back surface velocity from $u_{0}$ to $u_{m}$, as shown in Figure 3a. It happens that at a certain point, the tensile wave propagating inwards causes the fracture of the material and so, from the generation of two surfaces, two waves are also developed as shown in Figure 3b. One of those waves continues to go backwards into the medium, while the other remains trapped inside the spalled part of the specimen. The first pulse of the wave trapped in the spalled material towards the back surface leads to the increase of the velocity of the back surface and is generally referred to as the spall pulse because it indicates that the spall fracture has happened.

If we refer to the velocity of the back surface just before the arrival of the spall pulse as $u_{m}$, it is possible to define the difference between the peak back surface velocity $u_{0}$ and $u_{m}$ as ${\Delta u}_{\mathrm{pb}}$, which is usually called pull-back velocity. Adopting the acoustic approach and thanks to the work of Novikov et al. [42], the tensile stress just before spalling $\sigma_{\mathrm{sp}}$ can be evaluated through the following approximation:

(1) $$\sigma_{\mathrm{sp}}=\frac{1}{2}\rho_{0}c_{0}{\Delta u}_{\mathrm{pb}}$$

where $\rho_{0}$ and $c_{0}$ are, respectively, the density and the speed of sound in the undisturbed material. It is evident that this approximation becomes less accurate as the conditions in the spall plane become less similar to that of the undisturbed material, and in spite of that it is a useful way to make an evaluation of the spallation stress reached in the specimen.

In this context, the laser interferometers have become the standard measuring devices. In particular, two technologies are the most diffused which are called, respectively, VISAR (Velocity Interferometer System for Any Reflector) and PDV (Photonic Doppler Velocimetry). In [43,44,45] more details about the cited measurements systems are given, but here the pros and cons of both are briefly discussed. The VISAR is able to perform velocity measurements which are more detailed, both temporally and spatially, but it has the limitation that it needs a strong return signal, and hence for materials with a low reflectivity and that overcome a great deformation or fragmentation the output data can be very noisy. On the contrary, the PDV is able to work with a lower return signal, but it is characterised by a lower spatial and temporal resolution. For this reason, the interferometry is a very well-suited technique when the tested materials are metals, which generally are reflective and produce flat and solid spallation ejecta, but it might not give the expected results when dealing with brittle and less reflective specimens, e.g., graphite. This is why usually the laser interferometry is combined with high-speed photography methods such as pulsed laser photography or laser shadowgraphy [46,47].

Indeed, when a high-energy laser beam is correctly focused on a target it is able to induce pressures of several gigapascals, which are more than sufficient to produce shockwaves. In fact, the impact of the photons emitted by high-power lasers with the surface of the specimen leads to its immediate heating. The plasma generated from the laser–matter interaction is extremely dense and energetic and will eventually tend to expand itself, inducing a strong pressure field on the surrounding matter.

Due to the complexity of the phenomena involved, the common approach to predict laser–matter interaction is through radiation hydrocode simulations, which according to the characteristics of the laser beam and the properties of the material, define the energy deposition map [30,48,49].

## 3. Numerical Simulation Case Study

The dynamic response of brittle materials is a subject of interest in various applications. In this sense, data from experiments in compression are easily available in the literature. However, the literature concerning the spall tensile behaviour of highly porous materials such as graphite is limited [26,50]. For this reason, the research work developed at the CEA research institute [51,52,53,54,55] is clearly relevant. In particular, the results of the experimental campaign conducted at the Luli2000 facility [28] enriched the knowledge concerning the spall fragmentation of graphite impacted by high-power lasers. During the aforementioned experimental research, a total number of 16 shots were fired on EDM3 graphite. From the 16 shots on graphite, 7 were identified as shots representative of four different damage regimes, meanwhile the other 2 shots were classified as threshold cases between two damage regimes. The ranking of the regimes goes from D1 to D4 as the severity of the damage increases. In Table 1, the shots and the respective characteristics of both the target and the laser beam are summarised. With the aim to ease the comparison between the data collected in the CEA research and those presented in this paper, the shot numbering is the same.

The ablation pressures reported in Table 1 have been evaluated through the Grün formula defined as follows [31]:

(2) $$P_{ab}=1440{\left(0.8\frac{I_{m}}{10^{5}}\right)}^{0.8}$$

where $P_{\mathrm{ab}}$ is the ablation pressure in GPa and $I_{m}$ is the laser intensity in ${\mathrm{GW}/\mathrm{cm}}^{2}$.

It is necessary to clarify that the equation has been validated for aluminium targets, but it has proven to deliver good first approximation values for graphite [54].

As highlighted in Table 1, the shots were performed with two distinct beams called North (NB) and South (SB). The two beams had the same energy source but were equipped with two distinct amplification chains. This layout allowed a greater quantity of shots per day, but the spatial intensity profile of the beams was inevitably different. For this reason, shots performed with the same amount of energy generated different conditions with the NB and the SB, and therefore the two series of shots need to be considered as realised with two different lasers [28]. This feature gives the further opportunity to benchmark with data coming from tests on two different lasers the approach developed in this work, underlining its reliability.

Since the typologies of commercially available graphite are many, it is convenient to give more insights about EDM3 graphite. EDM3 is a 22% porous graphite produced by POCO [56]. However, due to the paucity of details regarding this particular type of graphite, the authors decided to refer to the ATJ graphite which shows material properties almost identical to EDM3 [57].

Another important feature of the experimental conditions taken as the case study is that the back surface of the EDM3 graphite was covered with 1 μm of aluminium. The Al coating has the only scope of reflecting the laser diagnostic, hence increasing the VISAR and PDV return signal. It is necessary to specify that this coating is too thin to have any structural or mechanical effect on the graphite specimen, and thus in the simulations developed in this work, the aluminium has not been modelled.

In the following section, the main features of the numerical simulations of the laser energy deposition and the subsequent shockwave propagation into graphite targets are explained in detail.

## 4. Numerical Approach

As already mentioned, in order to correctly analyse the phenomena taking place during a laser-driven shock event, it is convenient to divide the process into two phases that will lead to the use of two different tools. The first phase is the laser–matter interaction, which has been simulated with Helios [32], a one-dimensional radiation-hydrodynamics code specifically developed to study the evolution of plasmas generated by radiation sources. The second phase is the propagation of the shockwave through the target; hence the authors chose to develop a 2D axisymmetric simulation through LS-Dyna. In the following sections, the numerical approach is described both for the Helios and the LS-Dyna procedure with reference to the chosen case study.

### 4.1. Helios

The Helios software is able to describe how the energy delivered by the laser beam interacts with solid matter and the resulting thermodynamic state of the target. To correctly predict the laser action, Helios needs to know the wavelength of the light and the intensity profile of the laser beam versus time. To describe the material behaviour, an EOS and an opacity table are needed [32]. The Luli2000 laser beam wavelength can be adjusted so that different energy levels can be achieved [27]. However, during the aforementioned experimental tests, the wavelength was fixed at 532 nm [28].

The EOS used to model graphite is the SESAME 7833 table, which refers to dense carbon. The use of the dense carbon SESAME table for simulating laser–graphite interaction has already been validated in [52,54]. The opacity table format used inside Helios is called PrOpacEOS; even in this case, the table of carbon was chosen.

For the sake of a qualitative check, the ablation pressures obtained with Helios simulations have been compared to the values obtained thanks to Equation (2) and the agreement was good.

Typically, the amplification chain of high-energy lasers is focused through phase plates with the aim of generating a beam with an axisymmetrical and Gaussian shape. Indeed, the laser beam of the Luli2000 laser facility does not make an exception and the effective calibrated intensity spatial profile has been measured through a CCD camera and reported in [28]. In view of the data coming from the CCD measurements, it is possible to conclude that both the North and South beams can be treated as axisymmetrical with an effective radius of roughly 2 mm.

**Figure 4:**
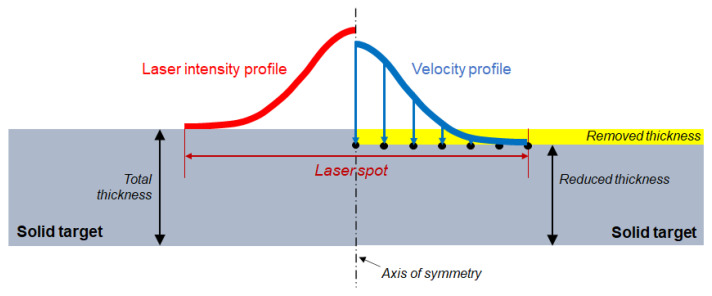
Schematic comparison between the actual phenomenon (**left side**) and the LS-Dyna model (**right side**).

Due to the critical importance of the laser shape, the authors decided to run a one-dimensional Helios simulation every 250 μm in radius to obtain the ablated plasma velocity spatial profile from the laser intensity spatial profile (Figure 4). In this way, the energy deposition produced by the laser can be transformed in a velocity time history, and it is possible to import it into LS-Dyna. The choice of using a velocity history rather than the pressure is linked to the fact that LS-Dyna has been built on an explicit integration scheme, and thus it performs better when dealing with imposed velocity rather than forces.

The results of the Helios simulations give fundamental information about the depth at which the material changes phase, becoming liquid or even plasma, and about the evolution of the shockwave through the target. The data evaluated with Helios can be used to define the correct initial conditions for the successive LS-Dyna simulation.

### 4.2. LS-Dyna

In LS-Dyna, a 2D axisymmetric representation of the target has been made, as shown in Figure 4. This type of geometry gives the best compromise between computational cost and level of detail of the results.

The radius of each modelled specimen was 4 mm, while the thickness varied according to the target thickness of the different shots and to the data coming from Helios. In fact, since the phenomena that take place during the laser deposition phase can only be simulated in Helios and are not reproducible with LS-Dyna, the FEM simulation has to start after the deposition phase. Indeed, the shockwave produced by the laser–matter interaction travels until a certain depth during the laser action, and hence the LS-Dyna model has to neglect the thickness travelled by the shockwave in the first 5 ns as shown in Figure 4. For this reason, this thickness is removed from the total thickness of the samples in LS-Dyna. This removed part always ablates or at least melts, whereas the aim of the LS-Dyna simulation is that of reproducing the behaviour only of the solid part.

In this physical problem, the mesh size should be chosen in agreement with the sound speed of the material: given that the sound speed in graphite is about 2200 m/s and that the shockwave duration is due to the deposition time (i.e., 5 ns), the mesh should be fine enough to correctly predict this extremely short event. Hence, a disturbance that travels at the graphite speed of sound moves roughly 11 μm in 5 ns. For this reason, the mesh size in the axial direction was fixed at 10 μm. Meanwhile, in the radial direction, the mesh was set at 10 μm for the first 2 mm, in which the elements just below the laser spot are placed, and at 20 μm for the rest of the target to achieve a lower computation time.

**Figure 5:**
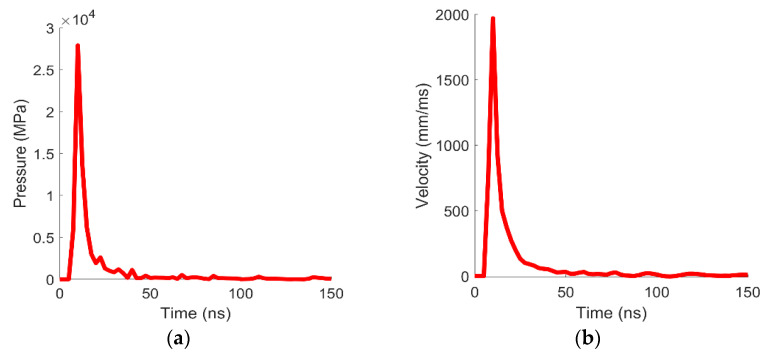
Pressure temporal profile (**a**) and velocity temporal profile (**b**) coming from Helios and applied as boundary condition in LS-Dyna. Notice that the applied pressure is still roughly 250 MPa at 140 ns.

As displayed in Figure 4, the velocity distribution coming from Helios is given as a displacement boundary condition on the nodes of the mesh. It is important to notice that even if the action of the laser is limited to 5 ns, the expansion of the plasma applies a relatively high pressure for almost 150 ns (see Figure 5); hence, it is necessary to take into account the velocity profile coming from Helios results at least for that amount of time.

#### Material Model

In view of the considerations made in the case study analysis, the reference material for graphite is the commercial ATJ graphite, which has a density of 1.68 g/cm^3^ [57,58]. This material is essentially brittle, but the porosity significantly increases the difficulty of describing its behaviour.

In the context of plate impacts and laser-induced shockwaves, the elastic–perfectly plastic material model proved to be reliable to characterise graphite [59]. Nonetheless, also in the VISAR measurements of the rear surface of the EDM3 graphite reported in [28], the wave seems to have the characteristic shape of a material which has overcome the Hugoniot elastic limit. Since graphite is effectively known as a fragile material, considering a plastic deformation may sound anomalous; however, its function is that of partially replicating the mechanism of closure of the pores. In fact, when the compressive shockwave travels in the graphite, it acts a pressure on the pores, which as a reaction tend to close. During the pores’ closure, some of the kinetic energy of the wave gets absorbed, resulting in a foam-like behaviour of the graphite. Thus, a partial analogy might be identified between the graphite pores’ closure and the plasticity typical of ductile materials in the kinetic energy absorption.

However, the perfectly plastic deformation did not provide all the kinetic energy absorption necessary to fit the experimental data and so a damping proportional to the stiffness was added.

Furthermore, the material model was completed with a spalling behaviour. The spallation value was tuned thanks to the work of Hébert et al. [59]; they proposed a spallation value for graphite of 140 MPa, which is almost twice as high as its static tensile strength. In LS-Dyna, the spallation is treated through a routine that checks if the elements reach a maximum principal stress equal to the spallation stress and when it happens the elements are flagged as spalled and they are no longer allowed to bear any further tensile loads [39]. The target should also be given the possibility to produce fragments, and therefore an erosion criterion on the maximum principal strain at failure was added to the material model.

The Gruneisen equation of state used to characterise the solid graphite shock behaviour was defined thanks to the shock Hugoniot data from the work of the Los Alamos Scientific Laboratory [58].

In Table 2, the characteristics of ATJ graphite proposed in this paper are summarised.

All the material properties described in the previous paragraph have been setup using the shot S7 as benchmark. Once the values were able to produce a reliable simulation of this shot, they were kept the same for all the other shots. The choice of this shot as reference is due to the fact that it has generated intermediate damage conditions on the back surface. In the following section, the results from the simulations of all the shots are presented and discussed.

## 5. Results

**Figure 6:**
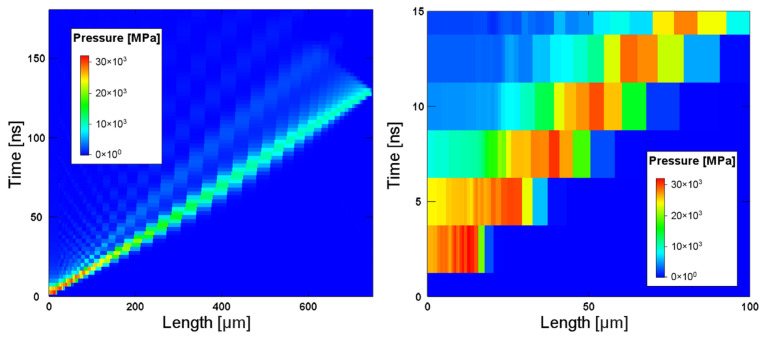
Representation of the pressure plots of the shot S7 used to find the shockwave front at the end deposition time. The figure on the right is the magnification of the left one.

Following the described approach, a simple procedure to find the shockwave front in Helios is represented in Figure 6. At 5 ns, i.e., at the time of the end of laser energy deposition, the front of the wave is at 35 μm from the front surface. In this case, the shot considered is the S7, but the procedure is the same for all the shots. This thickness cannot be ignored, because in the higher intensity shots, e.g., the S17, the depth reached by the shock front is almost 70 μm, which represents practically 10% of the total target thickness of 750 μm. The simulation examined for this analysis was always the one at the centre of the laser spot, and hence the one at higher intensity. Even if the hydrodynamic computation has been made every 250 μm in radius, the same depth was considered for all the laser spots. In Table 3 the depth reached for each shot and the resulting thickness of the LS-Dyna model are reported.

In the following, the first shots to be discussed will be those fired with the South beam, given that the shot on which the material properties have been calibrated is the S7.

### 5.1. South Beam Shots

The shots of the SB series are S7, S10, S15 and S17. Since the S10 was identified as a threshold between the damage regimes D1 and D2, it will be treated separately. Therefore, concerning the shots S7, S15 and S17, the target thickness was fixed to 0.75 mm and the laser intensity was increased; hence, in this section the same order of discussion will be followed.

**Figure 7:**
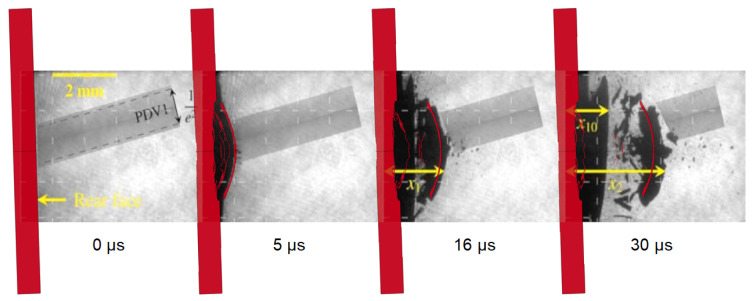
Comparison between numerical simulation and shadowgraphies of the shot S7 (reprinted from [28]).

In Figure 7, the comparison between the results of the LS-Dyna simulation of the shot S7 and the respective shadowgraphy sequence is highlighted. The shadowgraphy image at 0 μs allowed us to define the initial tilt of the target (i.e., about 2 degrees of rotation) and the axial position of the front surface of the graphite specimen not only for this shot, but for all the tests.

From Figure 7, it appears clearly that the radial size as well as the shape and speed of the ejected fragment is correctly predicted by the model. Indeed, due to the element erosion criteria implemented in the material model some debris behind the main fragment were deleted by the computation; however, the comparison between the thicknesses of the main fragment of the simulation and of the experiment is not easy with just the shadowgraphies. Probably, the second compact spalled object could not be simulated because of the erosion criteria. However, the higher displacement of the unbroken part of the experimental target might be partly associated with the displacement of the sample holder, which was not simulated in LS-Dyna.

Another important aspect can be highlighted by analysing Figure 7. In fact, the back surface of the target has a certain angle not only with the amplified camera for laser shadowgraphy, but also with the PDV (whose beam has been identified with a grey parallelepipedon in the shadowgraphies). This configuration implies that the PDV beam does not always point at the same spot on the back surface, which means that the speed data coming from this instrument will be regarding a moving spot on the back surface. Nonetheless, also when considering the same points on the surface, the velocity measured by the PDV is different from the exact axial direction of propagation of the debris. However, the PDV spectrogram reported in [28] is already corrected by the cosine of the resulting angle between the PDV beam and the vector normal to the back surface, except for shot S17 and N8. As a matter of fact, the debris cloud of those two shots is so chaotic that the velocity directions of the particles are totally different with each other, and hence no correction could have been made. In view of these considerations, the comparison between the PDV data and the simulation velocity results is not always straightforward, and hence the shadowgraphies are fundamental to properly understand the fragmentation mechanisms.

**Figure 8:**
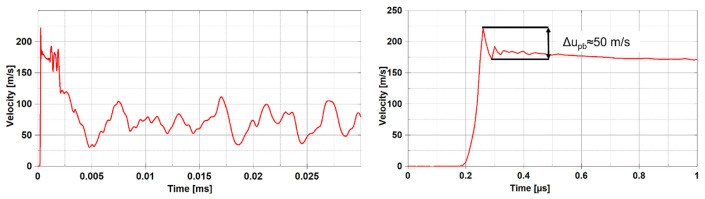
Axial velocity of the node at radius = 0 mm on the rear surface. The figure on the **right** is a portion of the **left** one.

On the contrary, analysing the velocities through the simulations is easier. For example, velocities can be evaluated even for only one node, as shown in Figure 8. This is the case of an ideal VISAR instrumentation with a beam that tends to a null radius. The node taken into account in the following is the one at the centre of the specimen (i.e., at radius 0 mm). By watching the temporal evolution of the axial velocity of that node, it is possible to identify the spall stress of the centre of the specimen according to Equation (1). In fact, given that the pullback velocity of that node is 50 m/s, using the acoustic approximation, the spall stress turns out to be of roughly 100 MPa. Nonetheless, it is clear that Equation (1) is quite inaccurate because, as already said, the spall value was fixed at 140 MPa in the material model. This discrepancy is mainly due to the fact that the analytical model neglects the change in density and in the speed of sound of the material, resulting in an erroneous evaluation of the spall value. Furthermore, another relevant effect is given by the damage mechanism of graphite which is almost never planar. It means that even though the central part of the specimen spalls, the broad parts are still attached to the specimen, and hence the central part velocity will be reduced.

From the previous considerations, it is evident how the application of laser interferometry measurements presents evident problems when it is applied to this type of experiment. However, the VISAR gives indispensable insights on the spallation behaviour of the materials. In fact, given the high temporal and spatial definition of VISAR, it is useful to evaluate the maximum velocity of the rear surface with extreme accuracy. In any case, it is clear that a satisfactory spall stress evaluation should be achieved numerically.

**Figure 9:**
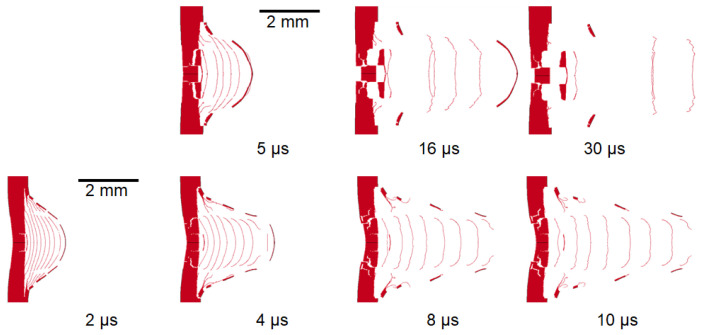
Numerical simulation sequencies of the shot S15 (**top**) and S17 (**bottom**).

In Figure 9, the sequences belonging to the simulation of shots S15 and S17 are displayed. In order to simplify the comparison with the shadowgraphies of the same shots reported in [28], the snapshots refer to the same temporal instants. Furthermore, the images belonging to the simulations have been opportunely cropped to fit into the pictures captured by the amplified cameras for laser shadowgraphy.

The images were taken at the same times between the shot S7 and the S15, and hence the juxtaposition can easily be conducted in this case. Indeed, in the S7, the main fragment is followed by few debris, meanwhile in the S15, the number of debris following the main fragment is evidently higher. Another difference concerns the perforation of the target, which did not happen in the shot S7, but it is clear in the image at 30 μs belonging to the shot S15, even though the two targets had the same thickness. Obviously, the increase in the damage of the rear surface is mainly linked to the increase of the laser intensity.

The same considerations can be made evaluating the shots S15 and S17. In fact, the S17 is characterised by a higher laser intensity, and hence an increased severity of the damage.

**Figure 10:**
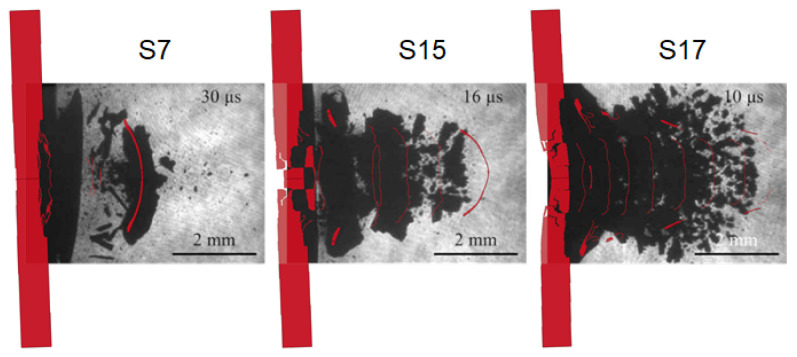
Representative shadowgraphies of shots S7, S15 and S17 (reprinted from [28]) and the respective numerical simulations.

With the aim of helping the reader find the resemblances between experiments and numerical simulations, the representative shadowgraphies of these three shots are compared to the respective numerical simulation instants in Figure 10.

As it is clearly visible, the shots S15 and S17 are also correctly predicted by this numerical approach both in the speed of the spalled surface and its shape. Nonetheless, it is evident that by increasing the laser intensity the behaviour of the resulting debris becomes progressively more chaotic, and hence the shape becomes harder to predict.

Concerning the three shots discussed until now, the PDV measurements are reported in [28]. Although the difficulties are already mentioned in the evaluation of debris velocities with PDV systems, in the following the comparison between the PDV experimental measurements and the velocities of the nodes on the rear PDV beam-like spot is given. The focus of this assessment is on the peak velocity and the average value of the debris flights after the detachment from the specimen. In Table 4, the data belonging to the velocities are reported.

As it can be easily noted from Table 4, the most successful simulation seems to be the S7. As a matter of fact, this was the reference shot with whom the simulation was calibrated, but this is not the only reason why that simulation is so effective. Although the PDV beam spot progressively moves from the exact centre of the specimen, it remains focused onto the main spalled debris as it can be seen in Figure 7. Hence, the PDV system certainly realises the same peak and average velocity measurement of the simulation. However, this is not true for the shots S15 and S17, whose PDV measurement collects information of different debris which have ever decreasing velocities, in spite of the simulation which always evaluates the speed of the centre spot of the specimen, which is obviously the fastest. Indeed, in this sense, average debris velocity data are always higher, meanwhile the speed data of the peak velocity coming from the simulation are more comparable to those coming from the VISAR measurements.

**Figure 11:**
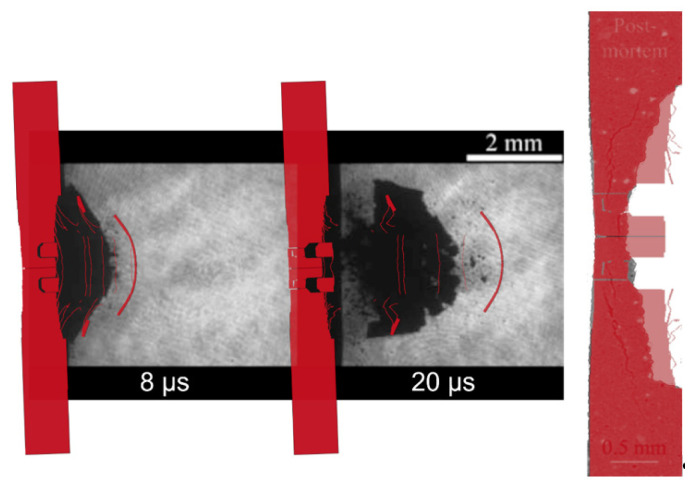
Shot S10 comparison between: numerical simulation results and shadowgraphies (reprinted from [28]) (**left**); post-mortem numerical simulation and tomography (reprinted from [28]) (**right**).

The shot S10 is treated separately not only because it is a threshold shot, and thus difficult to frame into a specific damage regime, but also because for this shot the tomography of the post-mortem specimen is available. In Figure 11, both the shadowgraphy and the tomography comparison is reported. In this case, even if the speed of the rear surface of the model is slightly higher than that showed by shadowgraphies, the shape is partially corresponding. Furthermore, the post-mortem specimen predicted by the model has the same radial dimension and the same depth of the experimental data. However, the tomography reveals a more regular and conical hole than the model. Another important similarity between the simulation and the real specimen concerns the front surface, which corresponds almost perfectly. Obviously, the thickness of the model is lower than that of the real sample because of the numerical approach used. This difference between the thicknesses will be more evident in the comparison of the shot N16 because the total thickness is lower.

### 5.2. North Beam Shots

Since the aim of this work is to demonstrate the reliability of the numerical approach also with different laser sources, in this section the results of the NB shots are discussed. In the case of the NB series, the logic followed was different, and in fact, the laser intensity was kept constant while the thickness was diminished. In this way, reducing the thickness resulted in a lower damping of the shockwave, and hence a more destructive effect on the rear surface. The shots are identified as N13, N16, N11, N9 and N8.

**Figure 12:**
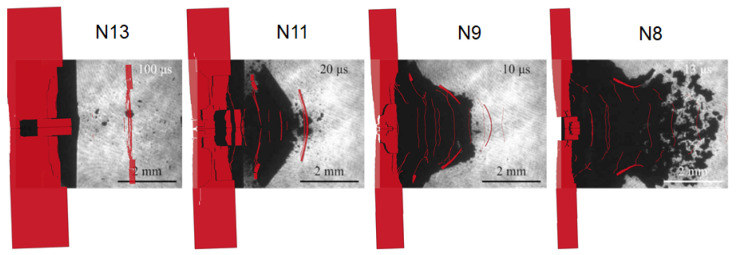
Numerical simulations of shots N13, N11, N9 and N8 and the respective shadowgraphies (reprinted from [28]).

In Figure 12, the benchmark between experimental and numerical shots has been reported. Except for some slight discrepancies with the shots N13 and N11, it appears clearly how the models are able to predict the fracture mechanism of the rear surface and the successive debris flight over a quite wide range of laser intensities. As a matter of fact, although the shape of the fractured plane of the shots N13 and N11 is not well reproduced by the models, it is true that the velocity of the faster debris is correctly followed by the debris of the simulation. Moving from the shot N11 to the N9, the aspect of the spalled surface resembles more a truncated cone than a cone. This truncated cone shape seems to be typical of the damage regime named D3, given that the debris of the shot N9 and S15 look very similar. However, the numerical model is able to properly remark the debris contour. The shot N8 is representative of the damage regime D4, and despite the chaotic trajectories of the debris particles, the simulation fittingly describes the debris cloud shape.

In Table 5, the comparison between the speed data from [28] and from the numerical simulations is reported. Almost all the considerations previously made for the SB shots can be made also for the NB shots. However, for the NB series, there was a representative shot for the damage regime D1, i.e., the N13. In view of the results of this shot, it clearly appears that the peak velocity is quite well predicted, while there are discrepancies on the average debris speed. This divergence can be understood by looking at the shadowgraphies comparison. As a matter of fact, the spalled surface descripted by the simulation is wider than the experimental data, and hence the speed averaged on the surface is higher.

**Figure 13:**
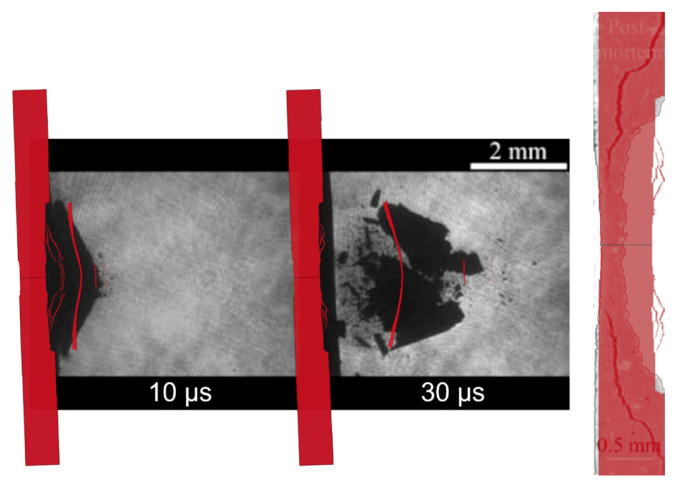
Shot N16 comparison between: numerical simulation results and shadowgraphies (reprinted from [28]). (**left**); post-mortem numerical simulation and tomography (reprinted from [28]). (**right**).

As for the case of the shot S10, also the shot N16 is treated after the others because it is a threshold shot and because its tomography is available. In Figure 13, the analogies between the experimental and numerical results for the shot N16 are shown. Notwithstanding the evident divergence of fragment shapes between the experimental and numerical spalled surface, the speed of the extreme part of the debris from the shadowgraphy can be compared to the little piece of debris of the simulation. Furthermore, the radial size of the post-mortem hole is similar, even though some differences can be found on the general shape of the hole. Indeed, as already anticipated, the difference between the thicknesses of the model and the real sample is more evident than in shot S10 since the N16 sample thickness is lower. The shape of the spot hit by the laser beam is well predicted by the numerical model.

## 6. Conclusions

In the present work, the shockwave behaviour of materials impacted by high-energy lasers beams was analysed. A laser beam is able to locally focus a great amount of energy on the surface of matter inducing high intensity shockwaves inside the material. For this reason, laser-induced shockwave tests could become an alternative for the testing of Beam Intercepting Device novel materials for high-energy particle physics applications.

In the first part of the paper, the fundamentals concerning shocks were shortly reviewed with a focus on laser-induced shockwaves. In particular, the wave generation, propagation and attenuation, as well as the successive reflection were discussed. The discussion covered also the methods to evaluate the spalling mechanism, which is a consequence of the shock reflection on a free surface.

The aim of this work was to develop a reliable numerical approach to predict the behaviour of materials impacted by laser beams with a particular attention to carbon-based materials such as graphite.

Accordingly, the proposed ‘two-step’ numerical model was explained in detail. The first step of this process is the simulation of the laser–matter interaction with the 1D radiation-hydrocode HELIOS. The results of the hydrodynamic simulation are then imported as boundary conditions for the successive 2D Finite Element LS-Dyna model. The FEM technique is used to reproduce the shockwave propagation into the target and the consequent spalling of the rear surface.

In order to test the numerical approach with literature-available experimental data, the testing campaign on EDM3 graphite conducted at the Luli2000 laser facility by the CEA research institute was taken as reference. This experimental campaign is considerably useful for the purpose not only because the combinations of target thicknesses and laser intensities were numerous, but also because the shots were fired with two different laser beams, hence giving the chance of assess the numerical approach reliability in a multitude of conditions. Thanks to the great amount of collected experimental data coming from PDV, VISAR and high-speed imaging, different comparisons could be performed.

One of the experimental shots was chosen as the calibration condition for some of the unknown material parameters including the damping values. Once the parameters were evaluated, they were kept constant for all the other simulations/testing conditions.

The results of the simulations reproducing the CEA testing campaign were compared with the experimental data coming from the shadowgraphies, tomographies, as well as the PDV and VISAR measurements. From the comparison, it clearly appears that the simulations were able to reasonably predict the shape, dimensions and residual speed of the debris in most of the cases, despite the limitations of a bidimensional representation of the phenomenon and of the erosion criteria used.

Furthermore, the spalled surface velocities acquired by the numerical models are also consistent with PDV and VISAR data, notwithstanding the remarkable speed and randomness of the events along with the difficulties regarding the measurement techniques for these tests.

Lastly, comparing the tomographies of the post-mortem specimens with the simulations, the proposed numerical approach is able to describe the front and back surface de-formations and damages to a satisfying level.

The description of the damage of the front surface is an estimation of the capability of the proposed method to reproduce the plasma production phase; the damage of the back surface is the result of the modelled propagation phase inside the material and of the dynamic fracture mechanisms.

The proposed methodology will be helpful in the postprocessing of future experiments to tune material model parameters starting from the experimental evaluations.

## Figures and Tables

**Table 1:** Essential data on reference shots (adapted from [28]). t is the target thickness, E is the total delivered laser energy, *I_m_* is the peak laser intensity, *P_ab_* is the ablation pressure evaluated through the Grün formula [31].

Laser Beam	Shot	t (mm)	E (J)	$I_{m}$ $\left(TW/cm^{2}\right)$	$P_{ab}$ (GPa)	Damage Regime
SOUTH BEAM	S10	1	235	1.72	46.7	D1–D2
	S7	0.75	121	0.89	27.4	D2
	S15	0.75	259	1.89	50.4	D3
	S17	0.75	547	4	91.7	D4
NORTH BEAM	N13	2	564	3.11	74.9	D1
	N16	0.75	167	0.92	28.3	D1–D2
	N9	1	636	3.50	82.5	D2
	N8	0.75	652	3.59	84.2	D3
	N11	1.5	608	3.35	79.6	D4

**Table 2:** ATJ graphite proposed material properties.

$\mathrm{Density}\;\left[\mathrm{kg}⁄m^{3}\right]$	1.768
$\mathrm{Young}\;\mathrm{Modulus}\;E\;\left[\mathrm{MPa}\right]$	11,500
$\mathrm{Yield}\;\mathrm{stress}\;\left[\mathrm{MPa}\right]$	102.5
$\mathrm{Spall}\;\mathrm{tension}\;\left[\mathrm{MPa}\right]$	140
$\mathrm{Bulk}\;\mathrm{sound}\;\mathrm{speed}\;\left[m/s\right]$	2200
$S_{1}\;\mathrm{Gruneisen}\;\mathrm{parameter}$	1.55

**Table 3:** Helios shot results concerning the shockwave depth reached at the end deposition time and the resulting target specimen after the deduction of the removed thickness.

Shot	Shockwave Depth [μm]	FEM Model Thickness [μm]
S7	35	715
S10	50	950
S15	50	700
S17	70	680
N13	62	1938
N11	63	1437
N16	35	715
N9	68	932
N8	68	682

**Table 4:** Comparison between the speed data of the PDV and VISAR measurements from (adapted from [28]) and of the numerical simulations.

Shot	Peak Velocity [m/s]			Average Debris Velocity [m/s]	
	Exp. (PDV)	Exp. (VISAR)	Simulation	Exp. (PDV)	Simulation
Shot S7	150	169	145	75	70
Shot S15	250	272	324	85	250
Shot S17	400	564	558	100	517

**Table 5:** Comparison between the speed data of the PDV and VISAR measurements (adapted from [28]) and of the numerical simulations.

Shot	Peak Velocity [m/s]			Average Debris Velocity [m/s]	
	Exp. (PDV)	Exp. (VISAR)	Simulation	Exp. (PDV)	Simulation
Shot N13	75	100	94	15	60
Shot N11	140	163	169	80	123
Shot N9	250	332	355	100	225
Shot N8	360	511	486	100	391

## Data Availability

The data presented in this study are available on request from the corresponding author. The data are not publicly available since they are part of an ongoing research project.
